# Functional Relevance of Improbable Antibody Mutations for HIV Broadly Neutralizing Antibody Development

**DOI:** 10.1016/j.chom.2018.04.018

**Published:** 2018-06-13

**Authors:** Kevin Wiehe, Todd Bradley, R. Ryan Meyerhoff, Connor Hart, Wilton B. Williams, David Easterhoff, William J. Faison, Thomas B. Kepler, Kevin O. Saunders, S. Munir Alam, Mattia Bonsignori, Barton F. Haynes

**Affiliations:** 1 Duke Human Vaccine Institute, Duke University School of Medicine, Durham, NC 27710, USA; 2 Department of Medicine, Duke University School of Medicine, Durham, NC 22710, USA; 3 Department of Immunology, Duke University School of Medicine, Durham, NC 22710, USA; 4 Department of Microbiology, Boston University School of Medicine, Boston, MA 02215, USA; 5 Department of Mathematics and Statistics, Boston Univeristy, Boston, MA 02215, USA; 6 Department of Surgery, Duke University School of Medicine, Durham, NC 27710, USA; 7 Department of Pathology, Duke University School of Medicine, Durham, NC 27710, USA

## Abstract

HIV-1 broadly neutralizing antibodies (bnAbs) require high levels of activation-induced cytidine deaminase (AID)-catalyzed somatic mutations for optimal neutralization potency. Probable mutations occur at sites of frequent AID activity, while improbable mutations occur where AID activity is infrequent. One bottleneck for induction of bnAbs is the evolution of viral envelopes (Envs) that can select bnAb B cell receptors (BCR) with improbable mutations. Here we define the probability of bnAb mutations and demonstrate the functional significance of key improbable mutations in three bnAb B cell lineages. We show that bnAbs are enriched for improbable mutations, which implies that their elicitation will be critical for successful vaccine induction of potent bnAb B cell lineages. We discuss a mutation-guided vaccine strategy for identification of Envs that can select B cells with BCRs that have key improbable mutations required for bnAb development.

## Highlights

• HIV-1 broadly neutralizing antibodies are enriched with low-probability mutations
• Improbable mutations can be functionally critical for bnAb neutralization breadth
• Critical improbable mutations are high-value targets for selection with vaccines

## Graphical Abstract

**Figure:**
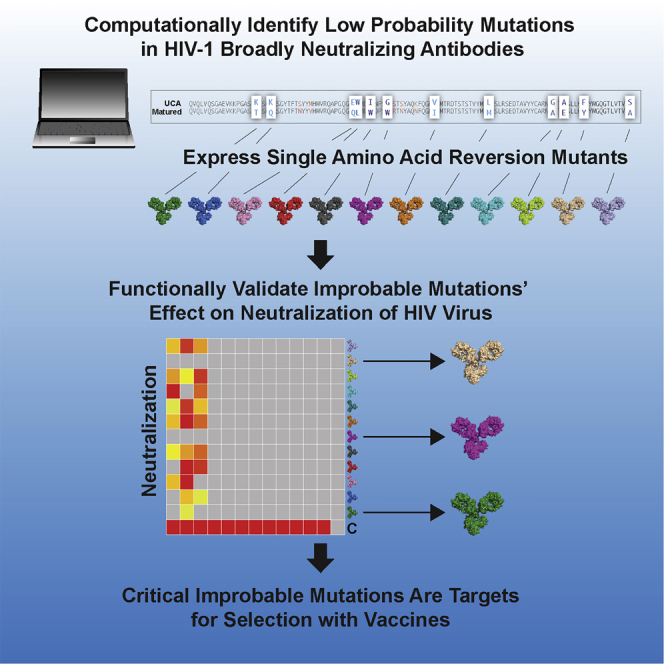

## Teaser

Not all mutations during B cell affinity maturation are equally probable. Wiehe et al. show that HIV-1 broadly neutralizing antibodies (bnAbs) are enriched with low-probability mutations and that these improbable mutations are often critical for HIV-1 bnAb neutralization breadth, thus making improbable mutations key targets for selection with vaccines.

## Main Text

The goal of HIV-1 vaccine development is the reproducible elicitation of potent, broadly neutralizing antibodies (bnAbs) (Haynes and Burton, 2017). BnAbs isolated from infected individuals have one or more unusual traits, including long third complementarity-determining regions (CDR3s) (Yu and Guan, 2014), autoreactivity (Kelsoe and Haynes, 2017), large insertions and deletions (Kepler et al., 2014a), and high somatic mutation frequencies (Burton and Hangartner, 2016). Somatic hypermutation (SHM) of the B cell receptor is the diversification method within the evolutionary process of affinity maturation that leads B cells to acquire high-specificity antigen recognition (Teng and Papavasiliou, 2007). Not all mutations acquired during antibody maturation are necessary for bnAb development; rather, high mutational levels may reflect the length of time required to elicit bnAbs (Georgiev et al., 2014, Jardine et al., 2016b). Consequently, shorter maturation pathways to neutralization breadth involving a critical subset of mutations are desirable, because antibody mutation levels induced by vaccines seldom reach the mutation frequencies observed in bnAbs (Easterhoff et al., 2017, Moody et al., 2011). Importantly, within this subset of critical mutations, some mutations may be probable and easy to elicit, whereas other mutations may be improbable and very challenging to elicit due to biases in how mutations arise during affinity maturation.

Somatic hypermutation occurs prior to antigen affinity-based selection during affinity maturation (De Silva and Klein, 2015, Victora and Nussenzweig, 2012). Somatic hypermutation is mediated by activation-induced cytidine deaminase (AID) (Di Noia and Neuberger, 2007), and AID preferentially targets specific nucleotide sequence motifs (“hot spots”), whereas targeting of other nucleotide motifs (“cold spots”) is disfavored (Betz et al., 1993, Pham et al., 2003, Yaari et al., 2013). AID initiates DNA lesions, and their subsequent repair results in a bias for certain bases to be substituted at the targeted position (Cowell and Kepler, 2000). The consequence of this non-uniformly random mutation process is that specific amino acid substitutions occur with varying frequencies prior to antigenic selection. Mutations at AID hotspots can occur frequently in the absence of antigen selection due to immune-activation-associated AID activity (Bonsignori et al., 2016, Yeap et al., 2015). Amino acid substitutions that occur infrequently generally require strong antigenic selection in order to arise during maturation (Brown et al., 1992, Kocks and Rajewsky, 1988). Such rare amino acid substitutions are improbable prior to selection for two reasons: 1) base mutations must occur at AID cold spots, and 2) due to codon mapping, multiple base substitutions must occur for a specific amino acid change. Within the critical subset of mutations that grant broad neutralization capacity to a bnAb lineage, those key mutations that are also improbable prior to selection may represent important events in bnAb maturation and are thus compelling targets for selection in a vaccine setting. We recently described a rare mutation, G57R, in DH270, a V3-glycan bnAb lineage, that conferred broad neutralization, thus demonstrating in one bnAb lineage that functionally important, improbable mutations can be roadblocks in HIV-1 bnAb development (Bonsignori et al., 2017). However, what has remained unclear is whether and to what extent such roadblocks are a general problem for bnAb elicitation. Here we describe the identification of improbable mutations in three bnAb B cell lineages and determine the functional relevance of these mutations for development of bnAb neutralization potency.

To determine the role of rare mutational events in bnAb development, we developed a computational program, “antigen receptor mutation analyzer for detection of low-likelihood occurrences” (ARMADiLLO), to identify improbable antibody mutations (see STAR Methods). We first applied ARMADiLLO retrospectively to the analysis of the G57R mutation in the DH270 bnAb lineage. ARMADiLLO estimated the G57R mutation to occur with < 1% frequency prior to selection. This mutation was functionally critical because reversion back to G57 in the lineage resulted in loss of heterologous neutralization (Bonsignori et al., 2017). Thus, the ARMADiLLO program can identify a known, key improbable mutation.

All BCR mutations arise prior to antigenic selection (Hwang et al., 2015). In HIV-1 infection, antibody heterologous breadth is not directly selected for during bnAb development because BCRs only interact with autologous virus Envs. Since improbable bnAb mutations can confer heterologous breadth, they represent critical events in bnAb development and make compelling targets for focusing selection with immunogens. To test this hypothesis, we analyzed three additional bnAb lineages with ARMADiLLO to identify improbable mutations (defined as < 2% estimated probability of occurring prior to selection; see STAR Methods) and then tested for their effect on neutralization during bnAb development. We chose three lineages that allowed for study of different levels of maturation in bnAb development: CH235, mid-stage bnAb development (Bonsignori et al., 2017); VRC01, late-stage bnAb development (Wu et al., 2015); and BF520.1, early-stage bnAb development (Simonich et al., 2016).

**Figure 1:**
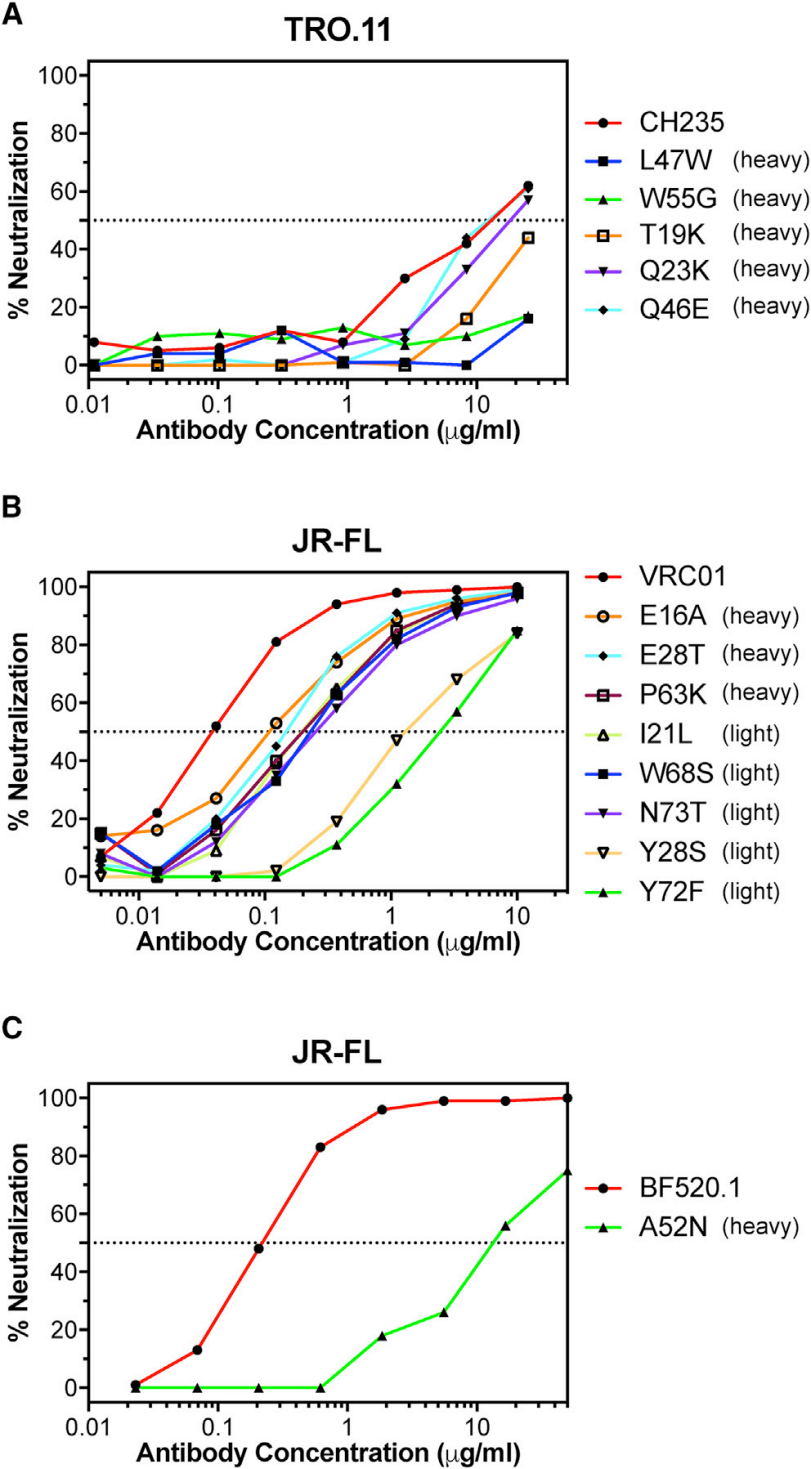
Improbable Mutations Confer Heterologous Neutralization in bnAb Development (A–C) BnAbs (A) CH235, (B) VRC01, and (C) BF520.1 and their corresponding mutants with reverted improbable mutations were tested for neutralization against heterologous viruses. The reversion of improbable mutations in all three bnAbs diminished neutralization potency. The chain in which the mutation was made is listed in parentheses. The estimated probabilities of the mutations, as well as the neutralization capacities of the antibody mutants against additional viruses, are included in Table S1.

CH235 is a CD4-binding-site (Gao et al., 2014) bnAb lineage that evolved to 90% neutralization breadth over 5 years of infection and acquired 45 V_H_ amino acid mutations (Bonsignori et al., 2016). We identified improbable mutations in the heavy chain of an early intermediate member of the lineage (also termed CH235), reverted each to their respective germline-encoded amino acids, and then tested antibody mutants for neutralization against heterologous, difficult-to-neutralize (tier 2) (Seaman et al., 2010), CH235-sensitive viruses (Figures 1A and S1A). Single amino acid reversion mutations resulted in either a reduction or an abrogation of neutralization for each of three improbable mutations—K19T, W47L, and G55W—demonstrating that improbable mutations in the CH235 lineage were indeed critical and could confer heterologous neutralization.

Interestingly, the K19T mutation was observed in all but one member of the CH235 bnAb lineage, and it was also present in two other CD4-binding-site bnAbs that were isolated from different individuals (Scheid et al., 2011) and shared the same VH gene segment (VH1-46) as CH235 (Figure S2A). We performed genomic sequencing of the individual from which the CH235 lineage was isolated and confirmed that K19T was a mutation and was not due to allelic variation in gene segment VH1-46 (Figure S2B). Structural modeling showed that the K19T mutation position was in close proximity to the N197 glycan site on the Env trimer (Figure S2C). The K19T mutation resulted in a shorter amino acid at this position, thus allowing for larger glycan forms at the heterogeneously glycosylated N197 position (Behrens et al., 2016) and providing a structural rationale for the effect of this mutation on heterologous breadth. Consistent with this hypothesis, CH235 neutralization of HIV-1 JR-FL, a tier 2 heterologous virus lacking the N197 glycan site, was the only CH235-sensitive virus tested that was unaffected by the T19K reversion mutation (Table S1 and Figure S1A). We introduced the K19T mutation into the CH235 UCA and observed improved binding to an early-autologous Env, which suggests that the improbable K19T mutation may have been selected for by an early autologous virus variant (Figure S2D). The presence of K19T in VH1-46-derived bnAbs from multiple individuals implicates this improbable mutation as a key developmental event shared among bnAb lineages within an epitope sub-class.

We next asked what role improbable mutations played in the maturation of a second CD4-binding-site-targeting bnAb lineage, termed VRC01, that acquired 43 V_H_ amino acid mutations (Zhou et al., 2010). We reverted improbable mutations in VRC01 and observed reduced potency of heterologous neutralization of HIV-1 JR-FL (Figure 1B), demonstrating that single improbable amino acid substitutions can also have functional consequences for heterologous neutralization capacity in a bnAb from late-stage lineage development. Improbable mutations in the light chain showed a larger effect on neutralization capacity than did heavy-chain mutations, further underscoring, along with an atypically short CDRL3 and a critical CDRL1 deletion (Zhou et al., 2013), the importance of key improbable events in the light chain for the development of the VRC01 lineage.

Babies develop bnAbs earlier after HIV-1 infection than adults (Goo et al., 2014, Muenchhoff et al., 2016). We analyzed the glycan-V3 epitope targeting BF520.1 bnAb, which was isolated from an HIV-1 infected infant and contained many fewer mutations (12 V_H_ amino acid mutations) compared to the VRC01 and CH235 lineages (Simonich et al., 2016). Reversion of N52A, an improbable mutation in the CDRH2, resulted in marked reduction in neutralization potency for all tier 2 BF520.1-sensitive viruses tested (Figures 1C and S1C; Table S1), suggesting that the acquisition of this improbable mutation may have played a key role in the early elicitation (< 15 months) of a bnAb with limited mutation frequency.

**Figure 2:**
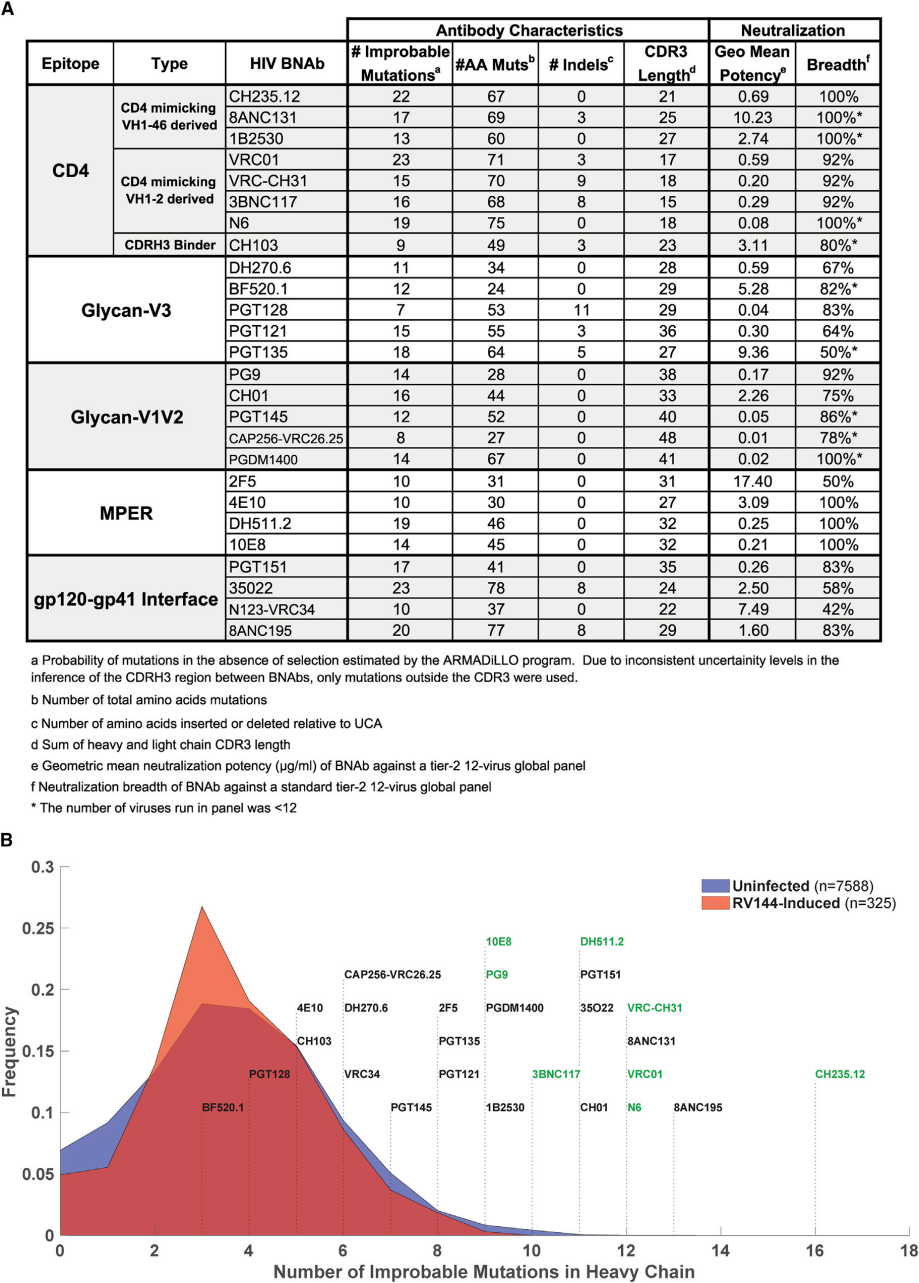
BnAbs Are Enriched For Improbable Antibody Mutations (A) Table of improbable mutations (at the < 2% cutoff level) for a representative set of bnAbs. (B) Distributions of the number of improbable mutations from antibody heavy-chain sequences from three groups: (1) “RV144-induced” antibodies were isolated from RV144 vaccinated subjects by antigenically sorting with RV144 immunogens (red shaded area); (2) “uninfected” antibodies correspond to duplicated NGS reads from IgG antibodies isolated from PBMC samples from 8 HIV-uninfected individuals (blue shaded area; see methods for details on sampling); and (3) a representative set of published bnAbs are shown labeled above dotted lines that correspond to their number of improbable mutations. Green labels indicate bnAbs with high potency (< 1 ug/mL) and breadth (> 90%).

While not all improbable mutations will be critical for bnAb development, a subset will be important, as demonstrated in the examples above. To provide a view of the scope of the problem for the development of many bnAb lineages, we estimated the number of improbable mutations for a representative set of bnAbs (Figures 2A, Table S2). Compared to Env-reactive antibodies induced by an HIV-1 vaccine candidate (Rerks-Ngarm et al., 2009) or antibodies isolated from HIV-1 uninfected individuals (Williams et al., 2015), the broadest and most potent HIV-1 bnAbs had the highest numbers of improbable mutations (Figure 2B). This result may follow directly from the observations that bnAbs tend to be highly mutated (Figure S3A; Burton and Hangartner, 2016), and the number of improbable mutations an antibody possesses is correlated with its mutation frequency (Figure S3B; Sheng et al., 2017). However, it is not known why most bnAbs are highly mutated. Recent work has shown that not all mutations in bnAbs are essential for neutralization activity (Jardine et al., 2016b). One hypothesis is that high mutation frequency is due to the extended number of rounds of somatic hypermutation required for a lineage to acquire a specific subset of mutations (Klein et al., 2013). If some of those specific mutations are also improbable, it is very likely that more probable mutations would be acquired prior to attaining key improbable ones. We found that for many bnAbs, the number of improbable mutations exceeded what would be expected by chance given their high mutation frequency (Figure S3C). This observation—along with our experimental observations demonstrating that a subset of improbable mutations are important for neutralization capacity—is consistent with the notion that improbable mutations may act as key bottlenecks in the development of bnAb neutralization breadth. Thus, during chronic HIV-1 infection with persistent high viral loads that are required for bnAbs with improbable mutations to develop (Gray et al., 2011), excess numbers of probable mutations also accumulate. Probable mutations arise easily from the intrinsic mutability of antibody genes, and unlike improbable mutations, they may not require Env selection (Bonsignori et al., 2016, Hwang et al., 2017, Neuberger et al., 1998). Thus, if the selection of critical improbable mutations can be targeted with Env immunogens, it should be possible to accelerate bnAb maturation to result in the induction of bnAb lineages with fewer mutations than those that occur in the setting of chronic HIV-1 infection.

Vaccine strategies aiming to select for specific maturation mutations in a clonal lineage first require adequate priming immunogens capable of engaging bnAb precursors. Recent progress has been made in the design of immunogens that bind bnAb UCAs (Jardine et al., 2016a, McGuire et al., 2013, Steichen et al., 2016, Zhang et al., 2016). Once UCAs are engaged, a vaccine must elicit mutations that keep antibody maturation on a pathway toward heterologous breadth. Immunization studies with fully germline-reverted bnAb-UCA-knockin mice have so far failed to demonstrate the acquisition of the specific mutations that lead to full bnAb neutralization capacity (Briney et al., 2016, Tian et al., 2016), suggesting that the pathways that lead to breadth are blocked and not circumvented with the current generation of immunogens. Based on our analysis here, one significant barrier could be the acquisition of important mutations that are highly improbable and thus infrequent prior to selection in the germinal center. Therefore, we propose a mutation-guided vaccine design and immunization strategy that harnesses the ability to identify functional improbable bnAb mutations using the ARMADiLLO program and antibody functional studies. This strategy would involve choosing the correct sequential Envs to precisely focus selection toward the most difficult-to-induce mutations, while allowing the key easier, more probable mutations to occur due to antibody intrinsic mutability from immune-activation-associated AID activity. Our proposed vaccine design strategy differs from the approach of targeting minimally mutated bnAbs (Jardine et al., 2016b) because it identifies the most rate-limiting mutations in bnAb development, therefore allowing for a higher level of precision with which to apply immunogen design.

Because improbable mutations arise either as rare intrinsic mutations or by selection by antigens derived from autologous virus, not all improbable mutations are required for mediation of heterologous neutralization (Table S1). Thus, while we showed that bnAbs are enriched for the number of improbable mutations relative to antibodies from uninfected or vaccinated individuals, work remains to functionally assess all of the improbable mutations to define those required for bnAb affinity maturation. Additionally, intrinsically mutable positions (Neuberger et al., 1998) can also be capable of conferring heterologous breadth. We identified one such functionally important probable mutation in the CH235 lineage, S57R (Table S1). However, such highly probable mutations should by definition arise frequently, and they are not likely to represent barriers in bnAb development. The W47L improbable mutation in the CH235 UCA had a negative effect on early autologous virus binding (Figure S2C), suggesting that it was selected by a later autologous virus and underscoring that mutation-guided vaccine strategies may require careful attention to the ordering of immunogens. While our analysis did not rule out that highly mutated non-neutralizing antibodies arising in chronic infection could also have large numbers of improbable mutations, such antibodies would not possess the critical improbable mutations that we propose should be targets for vaccine design.

Interestingly, bnAbs that had relatively small numbers of improbable single somatic mutations (Figure 2A) possessed other unusual antibody characteristics that were due to additional improbable events, such as indels (PGT128) or extraordinary CDR H3 lengths (CAP256-VRC26.25). Our analysis suggests that during the evolutionary arms race between virus and host, Env trimers have adapted to the typical (i.e., probable) neutralizing B cell responses. Consequently, the sites of broad vulnerability on the Env trimer can only be recognized by those B cells that have acquired highly atypical properties, including large numbers of improbable somatic mutations. The ARMADiLLO program and mutation-guided vaccine design strategy presented here should be broadly applicable for vaccine design for other mutating pathogens.

## STAR★Methods

### Key Resources Table

**Table:** 

REAGENT or RESOURCE	SOURCE	IDENTIFIER
**Antibodies**		

Human Monoclonal CH235	(Gao et al., 2014)	
Mutant Antibody CH235.T19K	This paper	N/A
Mutant Antibody CH235.L47W	This paper	N/A
Mutant Antibody CH235.W55G	This paper	N/A
Mutant Antibody CH235.Q23K	This paper	N/A
Mutant Antibody CH235.Q46E	This paper	N/A
Mutant Antibody CH235.R57S	This paper	N/A
Inferred antibody CH235.UCA	This paper	N/A
Mutant Antibody CH235.UCA+K19T	This paper	N/A
Mutant Antibody CH235.UCA+W47L	This paper	N/A
Mutant Antibody CH235.UCA+E46Q	This paper	N/A
Mutant Antibody CH235.UCA+K23Q	This paper	N/A
Human Monoclonal VRC01	(Zhou et al., 2010)	N/A
Mutant Antibody VRC01.E28T	This paper	N/A
Mutant Antibody VRC01.E16A	This paper	N/A
Mutant Antibody VRC01.P63K	This paper	N/A
Mutant Antibody VRC01.W68S	This paper	N/A
Mutant Antibody VRC01.Y72F	This paper	N/A
Mutant Antibody VRC01.N73T	This paper	N/A
Mutant Antibody VRC01.I21L	This paper	N/A
Mutant Antibody VRC01.Y28S	This paper	N/A
Human Monoclonal BF520.1	(Simonich et al., 2016)	N/A
Mutant Antibody BF520.1.A52N	This paper	N/A
Human Monoclonal CH01	(Bonsignori et al., 2011)	N/A
Human Monoclonal CH31	(Bonsignori et al., 2012)	N/A
Synagis	Catalent Biologics	N/A

**Bacterial and Virus Strains**		

HIV-1 Strain CH505 T/F	(Liao et al., 2013b)	N/A
HIV-1 Strain JR-FL	(Koyanagi et al., 1987)	N/A
HIV-1 Strain DU156.12	(Li et al., 2006)	N/A
HIV-1 Strain Q23	(Poss and Overbaugh, 1999)	N/A
HIV-1 Strain TRO.11	(deCamp et al., 2014)	N/A
HIV-1 Strain 246-F3_C10_2	(deCamp et al., 2014)	N/A
HIV-1 Strain X1632_S2_B10	(deCamp et al., 2014)	N/A
HIV-1 Strain 398-F1-F6_20	(deCamp et al., 2014)	N/A
HIV-1 Strain CNE8	(deCamp et al., 2014)	N/A
HIV-1 Strain X2278_C2_B6	(deCamp et al., 2014)	N/A
HIV-1 Strain CNE55	(deCamp et al., 2014)	N/A
HIV-1 Strain CH119.10	(deCamp et al., 2014)	N/A
HIV-1 Strain BJOX002000.03.2	(deCamp et al., 2014)	N/A
HIV-1 Strain 25710-2.43	(deCamp et al., 2014)	N/A
HIV-1 Strain Ce703010217	(deCamp et al., 2014)	N/A
HIV-1 Strain Ce1176_A3	(deCamp et al., 2014)	N/A
HIV-1 Strain 45_01dG5	(Wu et al., 2012)	N/A
HIV-1 Strain RHPA4259	(Ochsenbauer et al., 2012)	N/A
MLV-SVA (negative control)	This paper	N/A

**Biological Samples**		

Human PBMCs from the CH505 subject	Duke CHAVI-001 Protocol	N/A

**Chemicals, Peptides, and Recombinant Proteins**		

CH505.M5 d8 gp120 293F/Monomer	(Bonsignori et al., 2016)	CH505 M5 gp120
QuickChange II Lightning site-directed mutagenesis kit	Agilent Technologies	Cat #210518
Expi293 media	Invitrogen	Cat #A1435102
Expifectamine	Life Technologies	Cat #A14524
Protein A beads	Pierce	Cat #PI-20334
Vivaspin 15	Sartorius Stedim	Cat #VS15T22
DMEM	Invitrogen	Cat #11995-065
PEI	Polysciences Inc.	Cat #23966
Freestyle293 media	Invitrogen	Cat #12338-026
Vivaflow 50	Sartorius Stedim	Cat #VF05P2
Lectin beads	Vector Laboratories	Cat #AL-1243
MES	Sigma	Cat #M8250
Methyl-α-pyranoside	Sigma	Cat #M6882

**Critical Commercial Assays**		

Britelite Plus Reporter Gene Assay System	PerkinElmer	Cat #6016769

**Experimental Models: Cell Lines**		

Human cell line TZM-bl	NIH ARRRP	Cat #8129
Human cell line Expi293F	Invitrogen	Cat #14527
Human cell line FreeStyle 293F	Invitrogen	Cat #R790-07

**Oligonucleotides**		

A table of primers (Table S3) is included in Supplementary Items		

**Software and Algorithms**		

ARMADiLLO Program	This Paper	http://sites.duke.edu/armadillo/
Prism	GraphPad	
Cloanalyst Program	(Kepler, 2013)	
PyMOL Molecular Graphics System, Version 1.8	Schrodinger, LLC	http://www.pymol.org
FLASh	(Magoč and Salzberg, 2011)	
FASTx	(http://hannonlab.cshl.edu/fastx_toolkit/)	
BiaEvaluation software	Biacore/GE Healthcare	
MATLAB version R2017a	Mathworks	

### Contact for Reagent and Resource Sharing

Further information and requests for resources and reagents should be directed to and will be fulfilled by the Lead Contact, Kevin Wiehe (kevin.wiehe@duke.edu).

### Experimental Model and Subject Details

Malawian individual CH505, from whom the CH235 lineage was isolated and VH1-46 gene segment alleles determined by genomic sequencing, was enrolled in the Center for HIV/AIDS Vaccine Immunology (CHAVI) 001, nonblinded, nonrandomized, observational study protocol at a CHAVI clinical site in Malawi after informed consent was obtained under protocols approved by the Institutional Review Board of the Duke University Health System, the National Institutes of Health, and a clinical site review board in Malawi (Tomaras et al., 2011).

### Method Details

#### Analysis of the Probability of Antibody Mutations

##### Simulating the Somatic Hypermutation Process

Because AID targets hot spots according to their underlying sequence motifs, the probability of a mutation is sequence context dependent, making an analytical computation of the probability of a mutation in the absence of selection all but intractable. Instead, we take a numerical approach via simulation. Here, we estimate the probability of an amino acid substitution by simulating the somatic hypermutation (SHM) process and calculating the observed frequency of that substitution in the simulated sequences. The simulation proceeds as follows. Given a matured antibody nucleotide sequence, we first infer its unmutated common ancestor (UCA) sequence (Kepler, 2013, Kepler et al., 2014b). Next, the matured antibody nucleotide sequence is aligned to the UCA nucleotide sequence and the number of sites mutated, t, is computed. Starting with the UCA sequence, 1) the mutability score of all consecutive sequence pentamers is computed according to the S5F mutability model (Yaari et al., 2013). 2) The mutability scores for each base position in the sequence are converted into the probability distribution, Q, by:

[1] $$Q_{i}=\frac{C_{i}}{\sum_{i=1}^{L}C_{i}}$$

where $C_{i}$ is the mutability score at position i and L is the length of the sequence. 3) A base position, b, is drawn randomly according to Q. 4) The nucleotide n, at b, is substituted according to the S5F substitution model (Yaari et al., 2013), resulting in sequence $S_{j}$ where j is the number of mutations accrued during the simulation. The procedure then iterates over steps 1-4 until j = t. This results in a simulated sequence, $S_{t}$, that has acquired the same number of nucleotide mutations as observed in the matured antibody sequence of interest. If at any iteration during the simulation a mutation results in a stop codon, that sequence is discarded and the process restarts from the UCA sequence. This simulation procedure is then repeated to generate 100,000 simulated matured sequences. These nucleotide sequences are then translated to amino acid sequences. We note that the simulation relies heavily on the accuracy of the S5F mutability and substitution models and that those models were trained on a dataset of highly-fidelity next generation sequencing reads of 7 individuals. While we feel the S5F model accuracy is adequate for our simulations, the method described here can easily be adapted to use improved models of AID targeting and substitution should they become available.

##### Estimating the Probability of an Amino Acid Substitution

The estimate of the probability of any amino acid substitution *U*→Y at site i given the number of mutations t observed in the matured sequence of interest is then calculated as the amino acid frequency observed at site i in the set of simulated sequences X, according to:

[2] $$\hat{\text{P}}\left(X_{i_{U\to Y}}|UCA,\;t\right)=\frac{1}{N}\sum_{j=0}^{N}1\left(X_{ij}=Y\right)$$

where $X_{i}$ is the amino acid at site i which has the amino acid U in the UCA sequence mutating to amino acid Y in the matured sequence of interest, UCA is the UCA sequence, N is the number of simulated sequences, 1 is an indicator function for observing amino acid Y at site i in the jth simulated sequence. This estimate is for an amino acid substitution *in the absence of selection* and we use this probability as a gauge of how likely it is that a B cell would arise to have this mutation prior to antigenic selection. Amino acid substitutions that are the result of mutations that occur in AID hot spots will have high probabilities, occur frequently and a subset of the reservoir of B cell clonal members would likely have these mutations present prior to antigenic selection. Amino substitutions that are the result of cold spot mutations or require multiple base substitutions will be much less frequent and could represent significant hurdles to lineage development and these substitutions may require strong antigenic selection to be acquired during B cell maturation.

##### Improbable Mutations

The probability of a specific amino substitution at any given position is the product of two components. The first component is due to the bias of the AID enzyme in targeting that specific base position and the DNA repair mechanisms preference for substituting to an alternative base. Practically speaking, substitutions that require mutations at AID cold spots and/or result in disfavored base substitutions by DNA repair mechanisms are infrequent and thus improbable. The second component is the number and length of available paths through codon space to go from an amino acid encoded by the codon in the UCA to that of the codon for the substituted amino acid in the matured sequence. To illustrate this, we turn to a practical example: the TAT codon which encodes the amino acid, Tyr. From the TAT codon, 5 amino acids are achievable by a single nucleotide base substitution (C,D,F,H,N,S), 12 amino acids by two base substitutions (A,E,G,I,K,L,P,Q,R,T,V,W) and 1 amino acid (M) by three base substitutions. Without considering the bias of AID, the Y->M mutation starting from the TAT codon is inherently unlikely to occur because it requires three independent mutational events to occur within the same codon. By simulating the SHM process, ARMADiLLO captures the interplay of these two components and is able to estimate the probability of any amino acid substitution prior to selection by taking both components into account.

For this study, we have selected a cutoff of less than 2% probability to classify mutations as “improbable.” We chose this cutoff to reflect a frequency in which the expected number of mutations in a B cell clone in a single germinal center would be less than or equal to 1. In order to calculate the expected number of mutations, we used estimates of the number of clonally related B cells in a germinal center. The range of these estimates is ∼10-100 clonally related B cells for immunizations in mice with various protein antigens (Jacob et al., 1991, Tas et al., 2016). Thus, for a mutation that has 2% probability, the expected number of B cells with this mutation in a germinal center is 1 in 50, reflective of one mutation per clone per germinal center. To demonstrate the effects of different choices of cutoffs, we applied additional cutoffs of 1%, 0.1% and 0.01% when calculating the estimated numbers of improbable mutations in a representative set of bnAbs and these additional data are included in Table S2.

##### Calculating the Expected Number of Improbable Mutations

The number of improbable amino acid mutations, M, in an antibody sequence at a given probability cutoff can be estimated by applying [2] and enumerating over the entire amino acid sequence. For example, CH235.12 is estimated to have M = 16 improbable mutations in its heavy chain when improbable mutations are defined as amino acid substitutions with < 2% estimated probability. We estimate the probability of getting M improbable mutations or greater at a given amino acid mutation frequency, u, from the empirical distribution of the number of improbable mutations observed in sequences simulated to acquire T amino acid mutations, where T = u^∗^L and L is the length of the sequence. To calculate the empirical distribution of improbable mutations for each antibody sequence of interest, we first randomly draw 1000 sequences from an antibody sequence dataset generated from NGS sequencing of 8 HIV-1 negative individuals and infer the UCA of each sequence (Kepler, 2013, Kepler et al., 2014b). From these randomly sampled UCAs, we then simulate the SHM process using the same simulation procedure as detailed above and stop the simulation when each sequence acquires T amino acid mutations. This results in a set of 1000 simulated sequences each with an amino acid mutation frequency of u. The probability of observing M or greater improbable mutations in the absence of selection is then:

[3] $$\text{P}\left(\text{X}\geq \text{M}\right)=\frac{1}{N}\sum_{j=0}^{N}1\left(X_{j}\geq M\right)$$

where N is the number of simulations (here N = 1000), $X_{j}$ is the number of improbable mutations in the jth simulated sequence (calculated from [2] over all amino acid positions in the sequence) and 1 is an indicator function. Here we exclude the CDR3 sequence from our calculations of both M and u as the inference of the UCA has widely varying levels of uncertainty in the CDR3 region depending on the input matured sequence.

Standard methods for determining selection at an amino acid site typically rely on the measure ω which is the ratio of non-synonymous mutations to synonymous mutations at that position in a multiple sequence alignment of related gene sequences. Here, we avoid this measure of selection for two reasons. In many instances in this study we have only two sequences to compare, the UCA and the matured sequence. This does not provide the number of observations needed for ω to reliably indicate selection. In some case, where we do have multiple clonal members to align, the number of mutational events at a site is also not sufficiently large enough for ω to be reliable. Second, ω is calculated under the assumption that non-synonymous mutations are of neutral fitness advantage. Clearly, due to the sequence dependence of AID targeting this assumption is violated in B cell evolution. Instead, we employ the heuristic that amino acid mutations that are estimated to be improbable yet occur frequently within a clone are likely to have been selected for. While indicative of selection, this too can be misleading if mutations occur early in a lineage, are neutral and generate a cold spot or *colder* spot, thus making it less likely for the position to mutate again. Thus, it is apparent that much work remains on developing rigorous methods for measuring selection in B cell evolution. Our approach here is to treat improbable amino acid mutations as candidates for selection and to ultimately confirm the fitness advantage conferred by such mutations through experimentally testing their effect on virus neutralization and antigen binding.

##### Antibody Sequences from HIV-1 Negative Subjects

We utilized a previously described next generation sequencing dataset generated from 8 HIV-1 negative individuals prior to vaccination (Williams et al., 2015). Briefly, to mitigate error introduced during the PCR amplification, we split the RNA sample into two samples, A and B, and performed PCR amplification on each, independently. Only VDJ sequences that duplicated identically in A and B were then retained. This approach allowed us to be highly confident that nucleotide variations from germline gene segments that occurred in the NGS reads were mutations and not error introduced during PCR. We refer to this dataset as “uninfected.”

##### Antibody Sequences from RV144-vaccinated Subjects

We utilized a previously described set of antibody sequences (Easterhoff et al., 2017) isolated from subjects enrolled in the RV144 HIV-1 vaccination trial (Rerks-Ngarm et al., 2009). Antibody sequences were isolated from peripheral blood mononuclear cells (PBMC) from 7 RV144-vaccinated subjects that were antigen-specific single-cell sorted with fluorophore-labeled AE.A244 gp120 d11 (Liao et al., 2013a). We refer to this dataset as “RV144-immunized.”

##### Analysis of Improbable Mutations in BnAbs

Sequences of HIV-1 bnAbs were obtained either from NCBI GenBank or from the bNAber database(Eroshkin et al., 2014). For the comparison of improbable mutations for the representative set of bnAbs, improbable mutations were calculated using the ARMADiLLO program described above. UCAs were inferred using Cloanalyst (Kepler, 2013, Kepler et al., 2014b). While many bnAbs had multiple clonal lineage member sequences available, some bnAbs had no other members isolated. Because of this, only the single sequence of the matured bnAb was used in the UCA inference in order to provide equal treatment of all sequences. Because uncertainty in the UCA inference is highest for the bases in the CDR3 region, precise determination of some mutations in this region is not feasible and we therefore ignored the CDR3 region in our analysis of the representative set of bnAbs. In the simulations, we prohibited any mutations from occurring in the CDR3 region by setting the probability of AID targeting to 0 for each base in the CDR3. Neutralization data for the bnAbs was obtained through the CATNAP database (Yoon et al., 2015) and corresponds to neutralization in the global panel of 12 HIV-1 Env reference strains(deCamp et al., 2014). For the calculation of geometric mean neutralization, undetectable neutralization was set to 100 μg/ml. Breadth was reported for all viruses that were tested and for several bnAbs (8ANC131, 1B2530, N6, CH103, BF520.1, PGT135, PGT145, VRC26.25, PGDM1400) neutralization data was not available for all 12 viruses in the global panel.

#### Genomic Sequencing of the VH1-46 Gene Segment

To confirm that K19T was a mutation and not an allelic variant in subject CH505 from which CH235 clonal members were isolated, we sequenced CH505 IGHV gene segments according to a previously described experimental protocol for high throughput genomic sequencing of Ig gene segments which is detailed in Scheepers et al. (Scheepers et al., 2015). Briefly, genomic DNA was isolated from cryopreserved PBMCs using the Allprep DNA/RNA purification kit (QIAGEN # 80204). Each IGHV gene family (IGHV1-IGHV6) was amplified with 3 replicate PCR reactions (Table S3). A second PCR reaction was carried out to add the complete Illumina indexes (Nextera XT indexes; Illumina). Sequencing was performed using the Illumina MiSeq platform using 2x300bp read chemistry. Sequencing was performed using the Illumina MiSeq platform. A custom sequence analysis pipeline was used to analyze the sequencing data for identifying novel alleles. Forward and reverse reads were merged using FLASh (Magoč and Salzberg, 2011) and quality filtered using the FASTx toolkit (http://hannonlab.cshl.edu/fastx_toolkit/). Primers were trimmed such that only the V region of the merged read was retained and all resulting reads were then de-duplicated. All sequences with fewer than 10 reads were discarded. We then aligned all sequences to all known IGHV gene segments using a custom semi-global pairwise alignment program. Sequences that matched closest to any reference VH1-46 allele (01-03) were retained. Sequences that included stop codons, reflective of potential pseudogenes, were discarded. We then built a multiple sequence alignment of the remaining sequences and produced a sequence logo plot weighted by the number of read copies for each sequence. For the 19^th^ codon, 96% of reads matched the VH1-46^∗^01 reference AAG triplet encoding a lysine, consistent with homozygous lysine at this position (the remaining 4% of reads are consistent with the error level introduced during PCR amplification). The sequencing thus confirms K19T in the CH235 clone was indeed a mutation in the CH505 subject. A consensus of the translated reads was identical to the entire VH1-46^∗^01 reference allele demonstrating no non-synonymous polymorphisms indicating that at the protein level the CH505 subject was homozygous VH1-46^∗^01.

#### Antibody Site-Directed Mutagenesis

BF520.1 mutant antibody genes were synthesized by Genscript and recombinantly produced. Mutations into antibody genes for CH235, CH235.UCA and VRC01 mutants were introduced using the QuikChange II Lightning site-directed mutagenesis kit (Agilent Technologies) following the manufacturer’s protocol. Single-colony sequencing was used to confirm the sequences of the mutant plasmid products. A table listing the primers used for introducing mutations is included as a supplementary item (Table S3). The CH235.UCA sequence used in this study differed from the published and deposited CH235.UCA sequence (Bonsignori et al., 2016) by one amino acid in the light chain, containing a methionine at position 4 in the light chain.

#### Recombinant Antibody Production

Antibodies were recombinantly produced as previously described (Saunders et al., 2017). Expi293 cells were diluted to 2.5 million cells/mL in Expi293 media on the day of transfection. 293i cells were co-transfected with 400 μg of heavy chain plasmid and 400 μg of light chain plasmid using Expifectamine per the manufacturer’s protocol. Five days after transfection the cells were centrifuged and the cell culture supernatant was collected and filtered with a 0.8 μm filter. The cell-free supernatant was concentrated to approximately 50 mL total volume and incubated with protein A beads (ThermoFisher) overnight at 4°C. The protein A beads were centrifuged for 5 min at 1200 rpm in a Sorval tabletop centrifuge. The beads were re-suspended in 25 mL of PBS with 340 mM NaCl to wash them and pipetted into an empty plastic column. The antibody was eluted off of the beads with two elutions of 15 mL each of 10 mM glycine pH 2.4 150 mM NaCl. The pH was neutralized by adding 1M Tris pH8.0 to a final volume of 10%. The eluate was concentrated in a Vivaspin 15 and buffer exchanged into PBS with successive rounds of centrifugation.

#### Recombinant Env Expression

CH505 transmitted/founder variant M5 gp120 was recombinantly produced as previously described (Saunders et al., 2017). One mg of plasmid DNA per 1 l of cells was diluted in DMEM and mixed with PEI. PEI:DNA mixtures were added to cells for 4 h. 293F (Invitrogen) cells were subsequently washed and diluted to 1.25 million cells/mL in Freestyle293 media. (Invitrogen). The cells were cultured for 5 days and on the fifth day the cell culture media was cleared of cells by centrifugation and filtered with 0.8 μm filter (Nalgene). The cell culture was concentrated with a vivaflow 50 with a 10 kDa MWCO. The concentrated cell culture supernatant was rotated with lectin beads (Vistar Labs) overnight at 4°C. The beads were pelleted by centrifugation the next day and re-suspended in MES wash buffer. The lectin beads were washed twice and the protein was eluted with methyl- α -pyranoside. The protein was buffer exchanged into PBS and stored at –80°C.

#### HIV-1 Neutralization

Antibody neutralization was measured in TZM-bl cell-based neutralization assays as previously described (Sarzotti-Kelsoe et al., 2014, Saunders et al., 2017). Neutralizing antibody activity was measured in 96-well culture plates by using Tat-regulated luciferase (Luc) reporter gene expression to quantify reductions in virus infection in TZM-bl cells. TZM-bl cells were obtained from the NIH AIDS Research and Reference Reagent Program, as contributed by John Kappes and Xiaoyun Wu. Assays were performed with HIV-1 Env-pseudotyped viruses as described previously (Li et al., 2005).Test samples were diluted in DMEM cell culture media from a starting dilution of 1:10 (VRC01 mutants), 1:25 (CH235 mutants) or 1:50 (BF520.1 mutants) followed by 7 serial 3-fold dilutions and then pre-incubated with virus (∼150,000 relative light unit equivalents) for 1 h at 37°C before addition of cells. Following a 48 h incubation, cells were lysed and Luc activity determined using a microtiter plate luminometer and BriteLite Plus Reagent (PerkinElmer Life Sciences). Neutralization titers are the antibody concentration (IC_50_) at which relative luminescence units (RLU) were reduced by 50% compared to RLU in virus control wells after subtraction of background RLUs. CH235 and BF520.1 and selected mutants were assayed for neutralization using a global panel of 12 HIV-1 Env reference strains (deCamp et al., 2014). The combination of BNAbs CH01 and CH31 (Bonsignori et al., 2012) (1:1 mix) was used as a positive control antibody. Simian virus amphotropic Murine Leukemia Virus (MLV-SVA) was used as a negative control virus. Neutralization titers are reported as inhibitory concentrations (IC_50_) of antibody in which relative luminescence units (RLU) were reduced by 50% compared to RLU in virus control wells after subtraction of background RLUs of virus and reported in units of μg/ml.

#### Antibody Binding Measurements

Antibody binding to M5 (early autologous virus variant of CH505 T/F virus) delta8 gp120 (Bonsignori et al., 2016, Gao et al., 2014) was measured by surface plasmon resonance (SPR; BIAcoreS200, GE Healthcare) analysis. CH505.M5 gp120 (100ug/mL) was injected over antibody captured surface following capture of each mAbs on anti-human Ig Fc immobilized sensors as described earlier (Alam et al., 2013). Anti-human IgG Fc mab was immobilized at 8,000-11,000 RU (Response Unit) on a series S CM5 sensor chip (GE Healthcare) using standard amine coupling chemistry. Each mAb was captured at 300-500 RU and anti-RSV Palivizumab (Synagis) mAb captured on an adjacent flow cell was used as a control to subtract non-specific binding. Surface regeneration was done using Glycine, pH 2.0, following a 180 s injection of gp120 at a flow rate of 3ouL/min. Data analysis was performed using BiaEvaluation software (GE Healthcare).

#### Structural Modeling

The CH235-gp120 complex (Bonsignori et al., 2016) (PDB:5F9W) was superposed on to a structure of the BG505 SOSIP trimer structure (Stewart-Jones et al., 2016) (PDB:5FYL) to identify the neighboring residues in the gp120 interface to the CH235 K19T mutation in the context of a fully-glycosylated Env trimer. Structural modeling and visualization was performed using PyMOL version 1.8 **(**http://www.pymol.org),

### Quantification and Statistical Analysis

Statistical details of experiments can be found in the figure captions where applicable. Correlation coefficients were calculated using MATLAB.

### Data and Software Availability

The ARMADiLLO program for estimating the probability of antibody mutations prior to antigenic selection is available for download at http://sites.duke.edu/ARMADiLLO. More detailed information on the improbable mutations in a selected set of bnAbs is listed in an expanded table included in the Supplementary Items (Table S2).

## Supplemental Information

Document S1. Figures S1–S3 and Table S1

Table S2. Expanded Table of Estimated Number of Improbable Mutations in HIV-1 bnAbs; Related to STAR Methods

Table S3. List of Primers; Related to STAR Methods

Document S2. Article plus Supplemental Information
