# Supplementary material for: Functional Relevance of Improbable Antibody Mutations for HIV Broadly Neutralizing Antibody Development
Source: Cell Host Microbe. 2018 Jun 13;23(6):759–765.e6. doi: 10.1016/j.chom.2018.04.018 (PMC6002614; doi:10.1016/j.chom.2018.04.018)
Supplement: Document S1. Figures S1–S3 and Table S1 [file mmc1.pdf]

Cell Host & Microbe, Volume *23*

Supplemental Information

Functional Relevance of
Improbable Antibody Mutations for HIV
Broadly Neutralizing Antibody Development

Kevin Wiehe, Todd Bradley, R. Ryan Meyerhoff, Connor Hart, Wilton B. Williams, David Easterhoff, William J. Faison, Thomas B. Kepler, Kevin O. Saunders, S. Munir Alam, Mattia Bonsignori, and Barton F. Haynes

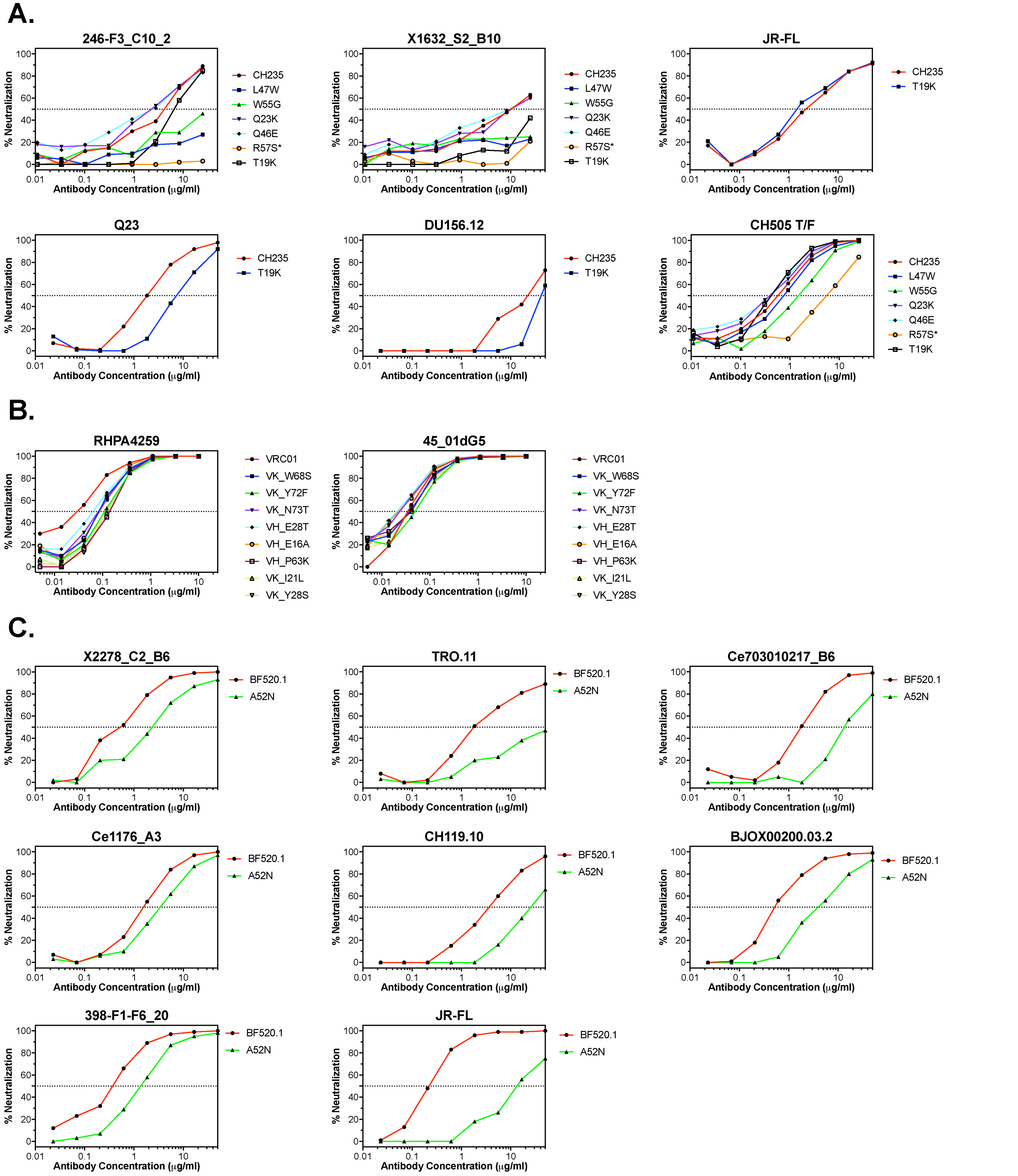

Figure S1. Neutralization of improbable mutation reversion mutants for CH235, VRC01, and BF520.1 (related to Figure 1).

Curves of the percent neutralization of WT (red line) **A)** CH235 **B)** VRC01 and **C)** BF520.1 and mutants containing reversions of identified improbable mutations against heterologous and autologous (CH505 T/F and 4501dG5 for CH235 and VRC01, respectively) viruses. 50% neutralization is denoted by a dotted line. Mutations probability estimates are shown in Table S1. One high probability CH235 mutation reversion, R57S, is also shown and denoted by an asterisk.

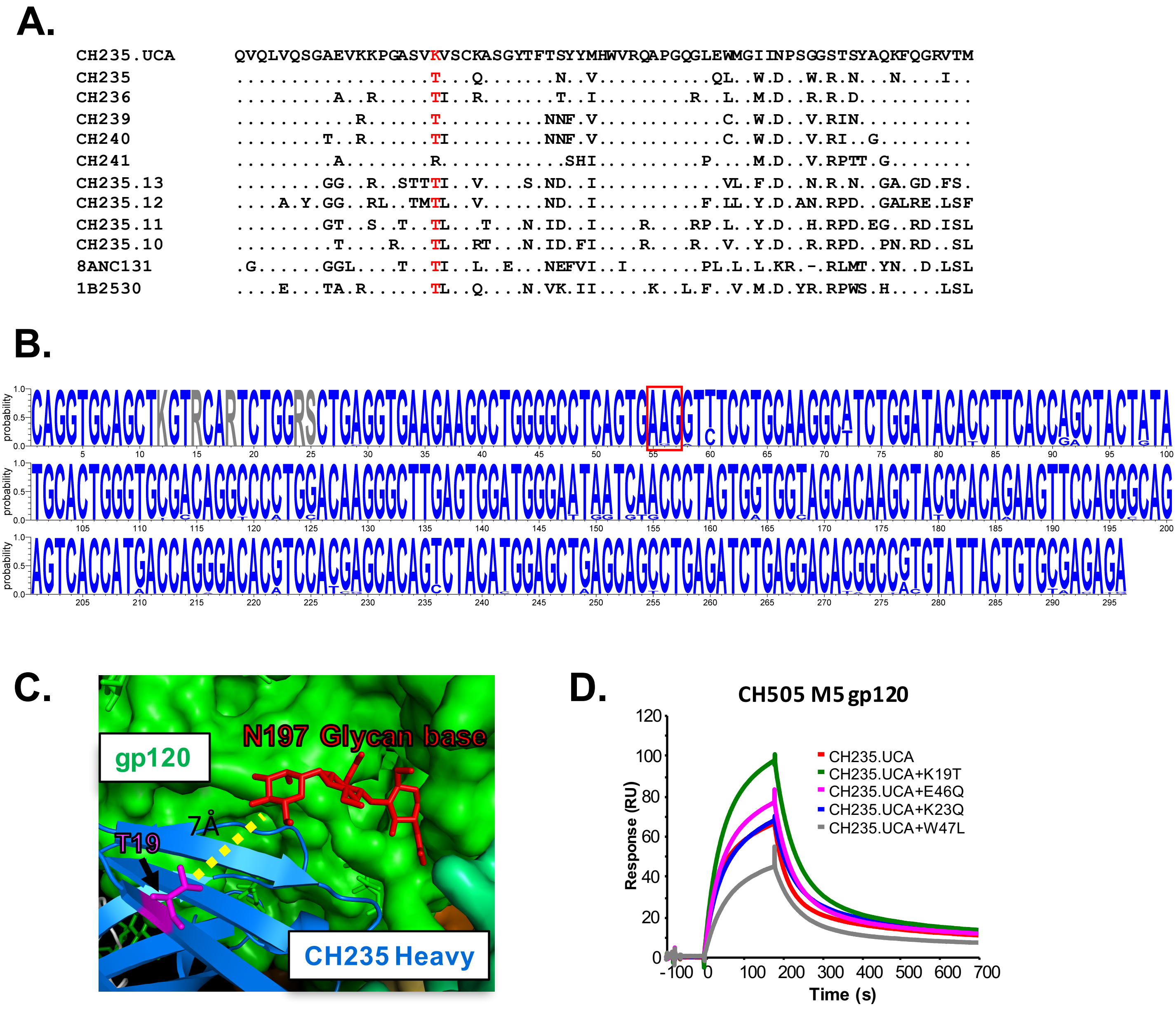

Figure S2. K19T mutation is conserved across all VH1-46 derived bnAb lineages and T19 position is proximal to N197 glycan site (related to Figure 1)

A) Amino acid multiple sequence alignment of the heavy chains of the three known VH1-46 gene segment-derived CD4 binding site bnAbs: 8ANC131, 1B2530, and the multiple member CH235 lineage aligned to the CH235 UCA. The K19T mutation (red) is observed in all three lineages suggesting convergence of this mutation in three distinct individuals. Dots denote an amino acid match with the CH235 UCA in that position. Only the first 70 amino acids of the sequence alignment are shown. **B)** Sequence logo plot of VH1-46 reads from genomic sequencing of the CH505 subject. No polymorphisms due to allelic variation were detected in the K19 codon (red box). Ambiguous bases in the primer region are denoted in gray. **C)** Superposition of the CH235 complex onto a fully glycosylated SOSIP trimer (5FYL) revealed that T19 (magenta) is in close proximity (7Å) to the N197 glycan base (red) resolved in the trimer structure (green). A longer Lys residue in the 19th position may sterically clash with larger glycans, providing a structural rationale for the conservation of the K19T mutation in VH1-46 derived CD4 binding site bnAbs. **D)** SPR sensorgrams for wildtype CH235 UCA and 4 UCA mutants containing improbable mutations show binding response to M5, a gp120 construct featuring a single amino acid mutation from the CH505 T/F that makes it more favorable for binding the CH235.UCA.

Table S1. Neutralization of bnAbs and mutants (Related to Figure 1)

A.

			Viruses																
	**Ab Mutant**	**Mutation Probability‡**	**CH505 T/F†**	**JR-FL**	**DU156.12**	**Q23**	**TRO.11***	**246-F3_C10_2***	**X1632_S2_B10***	**398-F1-F6_20***	**CNE8***	**X2278_C2_B6***	**CNE55***	**CH119.10***	**BJOX002000.03.2***	**25710-2.43***	**Ce703010217***	**Ce1176_A3***	**MLV SVA**
**CH235**	**WT**	-	**0.572**	**2.438**	**18.385**	**1.927**	**11.933**	**3.649**	**8.907**	>25	>25	>25	>25	>25	>25	>25	>25	>25	>25
	**T19K**	0.0116	**0.888**	**1.635**	>50	**7.39**	>50	**6.148**	>25	>25	>25	>25	>25	>25	>25	>25	>25	>25	>25
	**L47W**	0.0086	**0.699**	NT	NT	NT	>25	>25	>25	>25	>25	>25	>25	>25	>25	>25	>25	>25	>25
	**W55G**	0.0002	**1.663**	NT	NT	NT	>25	>25	>25	>25	>25	>25	>25	>25	>25	>25	>25	>25	>25
	**Q23K**	0.0161	**0.398**	NT	NT	NT	**19.869**	**2.641**	**11.821**	>25	>25	>25	>25	>25	>25	>25	>25	>25	>25
	**Q46E**	0.0185	**0.41**	NT	NT	NT	**12.142**	**2.178**	**7.125**	>25	>25	>25	>25	>25	>25	>25	>25	>25	>25
	**R57S**	0.2252	**6.102**	NT	NT	NT	>25	>25	>25	>25	>25	>25	>25	>25	>25	>25	>25	>25	>25
	**CH01+CH31**	-	**< 0.011**	**<0.023**	**3.078**	**<0.023**	**0.093**	**0.034**	**<0.011**	**0.039**	**0.059**	**0.025**	**0.035**	**0.468**	**0.977**	**0.16**	**< 0.011**	**0.018**	>25

B.

			Viruses			
	**Ab Mutant**	**Mutation Probability‡**	**JR-FL**	**45_01dG5†**	**RHPA4259**	**MLV-SVA**
**VRC01**	**WT**	-	**0.04**	**0.036**	**0.033**	>10
	**E28T (HC)**	0.0009	**0.141**	**0.019**	**0.069**	>10
	**E16A (HC)**	0.0012	**0.12**	**0.022**	**0.092**	>10
	**P63K (HC)**	0.0070	**0.209**	**0.038**	**0.133**	>10
	**W68S (LC)**	0.0007	**0.215**	**0.038**	**0.089**	>10
	**Y72F (LC)**	0.0090	**2.095**	**0.053**	**0.109**	>10
	**N73T (LC)**	0.0085	**0.261**	**0.025**	**0.088**	>10
	**I21L (LC)**	0.0095	**0.196**	**0.042**	**0.121**	>10
	**Y28S (LC)**	0.0010	**1.387**	**0.042**	**0.128**	>10
	**CH01+CH31**	-	**< 0.023**	**< 0.023**	**0.136**	>10

C.

			Viruses												
	**Ab Mutant**	**Mutation Probability‡**	**JR-FL**	**TRO.11***	**398-F1-F6_20***	**X2278_C2_B6***	**CH119.10***	**BJOX002000.03.2***	**25710-2.43***	**Ce703010217***	**Ce1176_A3***	**246-F3_C10_2***	**X1632_S2_B10***	**CNE8***	**CNE55***	**MLV-SVA**
**BF520.1**	**WT**	-	**1.315**	**1.866**	**0.39**	**0.467**	**3.894**	**0.598**	**40.449**	**1.901**	**1.472**	>0	>50	>50	>50	>50
	**A52N**	0.0005	**12.81**	>50	**1.247**	**2.238**	**22.203**	**3.971**	>50	**13.288**	**3.634**	>50	>50	>50	>50	>50
	**CH01+CH31**	-	**0.031**	**0.38**	**0.108**	**0.055**	**2.29**	**10.806**	**0.895**	**0.102**	**0.11**	0.124	0.078	0.183	0.113	>50

Neutralization (IC50 µg/ml)		
50 to 1	1 to .1	<0.1

*From global panel of 12 Tier-2 reference strains
† Autologous virus
‡ Estimated probability of mutation prior to selection as calculated by the ARMADiLLO program
NT=Not Tested

A.

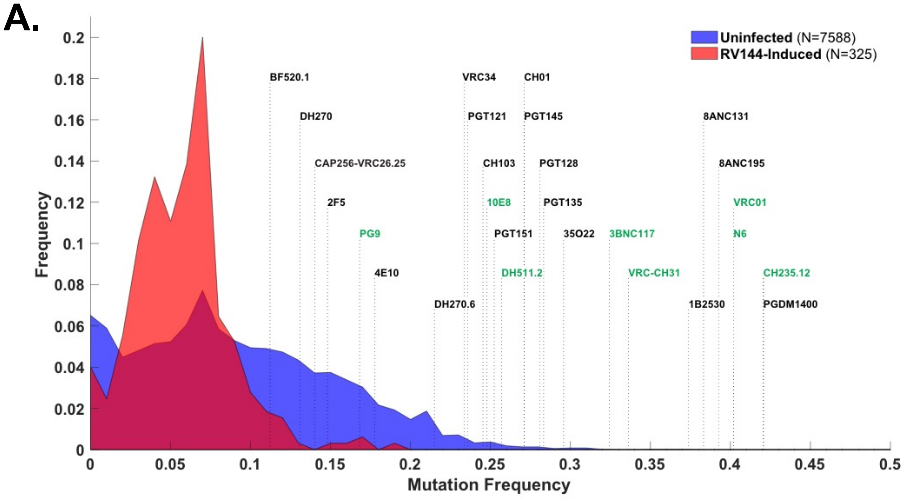

B.

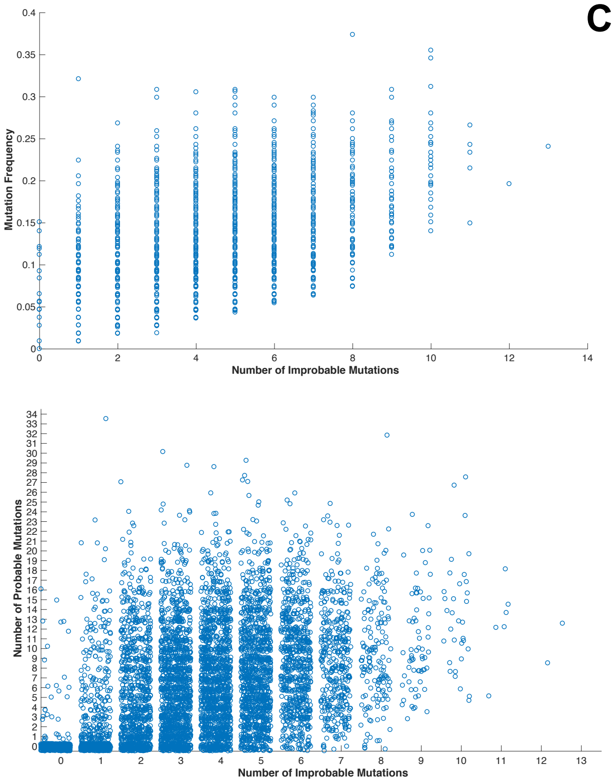

C.

BnAb	#AA mutations[A]	AA mut freq	# Improbable Mutations (M)[B]	Expected Frequency of >=M[C]	Counts
CH235.12*	45	42.1%	16	0.20	2/985
8ANC195*	44	39.3%	13	2.34	23/981
N6*	43	40.2%	12	4.35	43/989
VRC01*	43	40.2%	12	3.75	37/986
8ANC131*	41	38.3%	12	3.67	36/980
VRC-CH31*	39	33.6%	12	1.43	14/981
CH01*	29	27.1%	11	0.71	7/985
DH511.2*	28	25.7%	11	0.51	5/981
PGT151*	27	25.2%	11	0.62	6/974
35O22*	34	29.6%	11	1.32	13/984
3BNC117	36	32.4%	10	5.28	52/985
10E8*	27	24.8%	9	4.54	45/991
PGDM1400	45	42.1%	9	36.59	360/984
1B2530	40	37.4%	9	25.43	249/979
PG9*	18	16.8%	9	1.63	16/983
PGT135	32	28.3%	8	15.96	157/984
PGT121	25	23.6%	8	8.61	85/987
2F5*	16	14.8%	8	4.47	44/984
PGT145	29	27.1%	7	25.96	256/986
VRC34	25	23.4%	6	32.21	315/978
VRC26.25	15	14.0%	6	23.71	234/987
DH270.6	23	21.5%	6	29.67	292/984
CH103	26	24.5%	5	56.58	559/988
4E10	19	17.8%	5	46.97	465/990
PGT128	32	28.1%	4	82.36	808/981
BF520.1	12	11.2%	3	84.37	831/985

A Number of amino acid mutations, excluding CDRH3 region
B The number of improbable mutations M (at <2% cutoff) identified for the BNAb using ARMADiLLO
C The expected frequency of M or greater improbable mutations (at <2% cutoff) from simulations of SHM at the BNAb's AA mutation frequency
*Number of improbable mutations in this bnAb exceeds number expected by chance (cutoff=.05) given the mutation frequency of this bnAb

Figure S3. BnAbs have high mutation frequencies and mutation frequency is correlated with improbable mutations (Related to Figure 2)

A) Histograms of mutation frequency distributions from antibody heavy chain sequences from three groups: "RV144-induced" antibodies isolated from RV144 vaccinated subjects by antigenic sorting with RV144 immunogens (red shaded area); "Uninfected" antibodies correspond to duplicated NGS reads from IgG antibodies isolated from PBMC samples from 8 HIV-uninfected individuals (blue shaded area; see methods for details on sampling); a representative set of published bnAb antibody sequences are shown labeled above dotted lines corresponding to their mutation frequency (defined as total number of amino acid mutations in non-CDRH3 VDJ sequence divided by non-CDRH3 VDJ sequence length). Scatterplots of **B)** number of improbable mutations vs. amino acid mutation frequency (top panel) for 7588 NGS reads from IgG antibodies from PBMC samples from 8 HIV-uninfected individuals and number of improbable mutations vs. number of probable mutations (≥2%) (bottom panel). Number of improbable mutations was moderately correlated with number of probable mutations (Pearson's r=0.43). A stronger correlation was observed between improbable mutations and mutation frequency (Pearson's r=0.67) as expected because improbable mutations are a subset of the total amino acid mutations used to calculate amino acid mutation frequency. Jitter added in order to alleviate over-plotting in panel C. **C)** Number of improbable mutations expected by chance given a bnAb's mutation frequency (see STAR Methods section for details).
